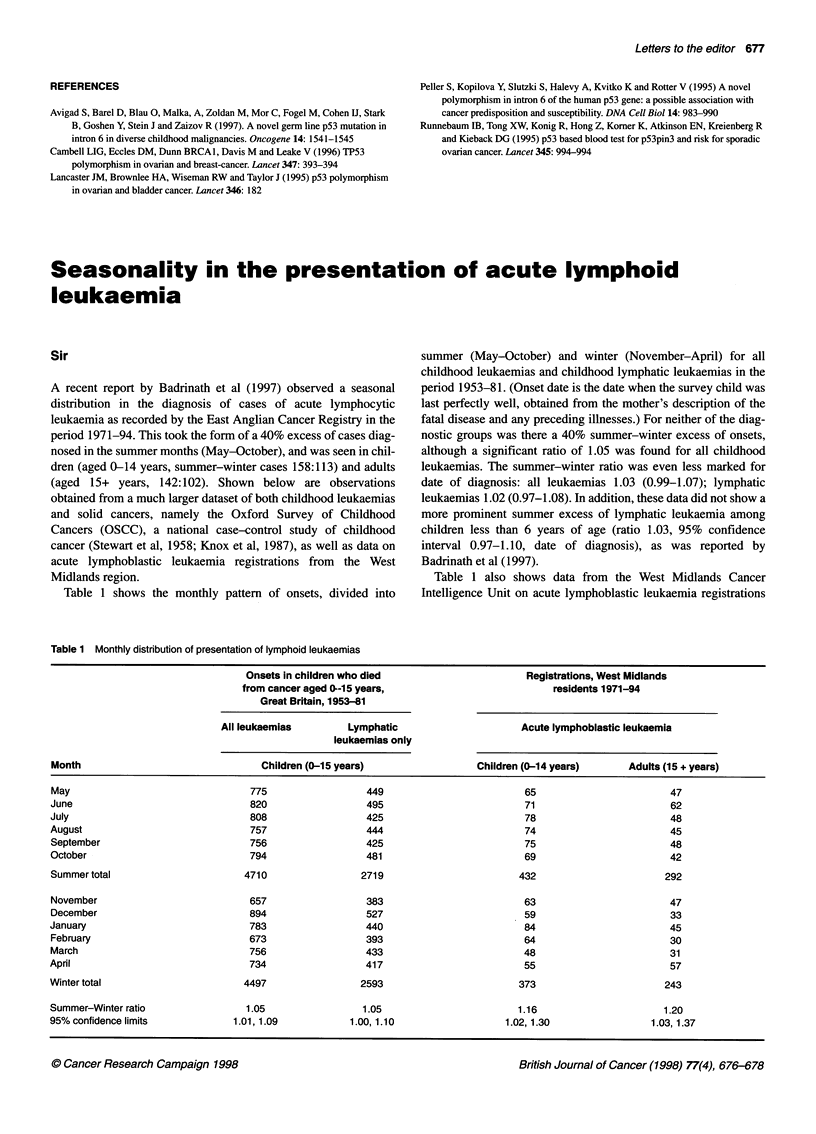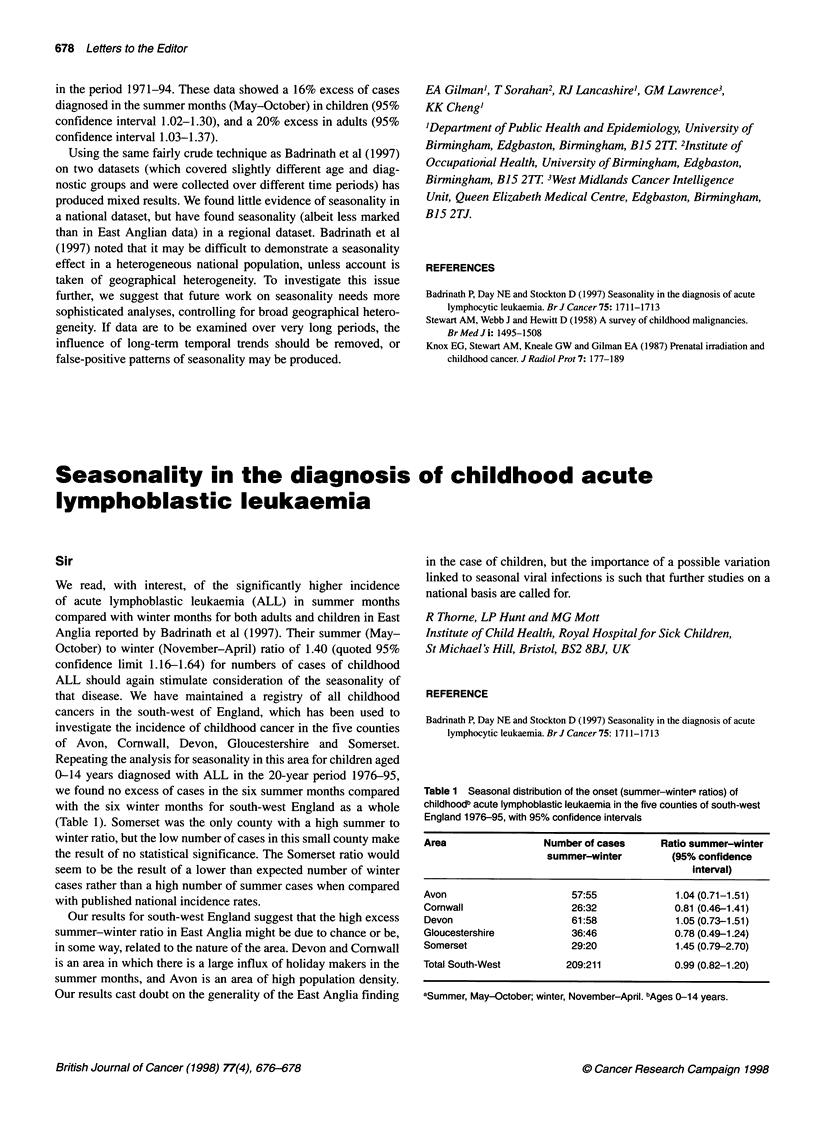# Seasonality in the presentation of acute lymphoid leukaemia.

**DOI:** 10.1038/bjc.1998.109

**Published:** 1998-02

**Authors:** E. A. Gilman, T. Sorahan, R. J. Lancashire, G. M. Lawrence, K. K. Cheng


### Seasonality in the presentation of acute lymphoid leukaemia

### Sir

A recent report by Badrinath et al (1997) observed a seasonal distribution in the diagnosis of cases of acute lymphocytic leukaemia as recorded by the East Anglian Cancer Registry in the period 1971–94. This took the form of a 40% excess of cases diagnosed in the summer months (May–October), and was seen in children (aged 0–14 years, summer–winter cases 158:113) and adults (aged 15+ years, 142:102). Shown below are observations obtained from a much larger dataset of both childhood leukaemias and solid cancers, namely the Oxford Survey of Childhood Cancers (OSCC), a national case–control study of childhood cancer (Stewart et al, 1958; Knox et al, 1987), as well as data on acute lymphoblastic leukaemia registrations from the West Midlands region.

Table 1 shows the monthly pattern of onsets, divided into summer (May–October) and winter (November–April) for all childhood leukaemias and childhood lymphatic leukaemias in the period 1953–81. (Onset date is the date when the survey child was last perfectly well, obtained from the mother's description of the fatal disease and any preceding illnesses.) For neither of the diagnostic groups was there a 40% summer–winter excess of onsets, although a significant ratio of 1.05 was found for all childhood leukaemias. The summer–winter ratio was even less marked for date of diagnosis: all leukaemias 1.03 (0.99–1.07); lymphatic leukaemias 1.02 (0.97–1.08). In addition, these data did not show a more prominent summer excess of lymphatic leukaemia among children less than 6 years of age (ratio 1.03, 95% confidence interval 0.97–1.10, date of diagnosis), as was reported by Badrinath et al (1997).

Table 1 also shows data from the West Midlands Cancer Intelligence Unit on acute lymphoblastic leukaemia registrations

**Table 1** Monthly distribution of presentation of lymphoid leukaemias

| | Onsets in children who died from cancer aged 0–15 years, Great Britain, 1953–81 | | Registrations, West Midlands residents 1971–94 | |
|---|---|---|---|---|
| | All leukaemias | Lymphatic leukaemias only | Acute lymphoblastic leukaemia | |
| Month | Children (0–15 years) | | Children (0–14 years) | Adults (15 + years) |
| May | 775 | 449 | 65 | 47 |
| June | 820 | 495 | 71 | 62 |
| July | 808 | 425 | 78 | 48 |
| August | 757 | 444 | 74 | 45 |
| September | 756 | 425 | 75 | 48 |
| October | 794 | 481 | 69 | 42 |
| Summer total | 4710 | 2719 | 432 | 292 |
| November | 657 | 383 | 63 | 47 |
| December | 894 | 527 | 59 | 33 |
| January | 783 | 440 | 84 | 45 |
| February | 673 | 393 | 64 | 30 |
| March | 756 | 433 | 48 | 31 |
| April | 734 | 417 | 55 | 57 |
| Winter total | 4497 | 2593 | 373 | 243 |
| Summer–Winter ratio | 1.05 | 1.05 | 1.16 | 1.20 |
| 95% confidence limits | 1.01, 1.09 | 1.00, 1.10 | 1.02, 1.30 | 1.03, 1.37 |

© Cancer Research Campaign 1998

in the period 1971–94. These data showed a 16% excess of cases diagnosed in the summer months (May–October) in children (95% confidence interval 1.02–1.30), and a 20% excess in adults (95% confidence interval 1.03–1.37).

Using the same fairly crude technique as Badrinath et al (1997) on two datasets (which covered slightly different age and diagnostic groups and were collected over different time periods) has produced mixed results. We found little evidence of seasonality in a national dataset, but have found seasonality (albeit less marked than in East Anglian data) in a regional dataset. Badrinath et al (1997) noted that it may be difficult to demonstrate a seasonality effect in a heterogeneous national population, unless account is taken of geographical heterogeneity. To investigate this issue further, we suggest that future work on seasonality needs more sophisticated analyses, controlling for broad geographical heterogeneity. If data are to be examined over very long periods, the influence of long-term temporal trends should be removed, or false-positive patterns of seasonality may be produced.

*EA Gilman[1], T Sorahan[2], RJ Lancashire[1], GM Lawrence[3], KK Cheng[1]*
*[1]Department of Public Health and Epidemiology, University of Birmingham, Edgbaston, Birmingham, B15 2TT. [2]Institute of Occupational Health, University of Birmingham, Edgbaston, Birmingham, B15 2TT. [3]West Midlands Cancer Intelligence Unit, Queen Elizabeth Medical Centre, Edgbaston, Birmingham, B15 2TJ.*

### REFERENCES

Badrinath P, Day NE and Stockton D (1997) Seasonality in the diagnosis of acute lymphocytic leukaemia. *Br J Cancer* **75**: 1711–1713

Stewart AM, Webb J and Hewitt D (1958) A survey of childhood malignancies. *Br Med J* **i**: 1495–1508

Knox EG, Stewart AM, Kneale GW and Gilman EA (1987) Prenatal irradiation and childhood cancer. *J Radiol Prot* **7**: 177–189